# Early Antenatal Prediction of Gestational Diabetes in Obese Women: Development of Prediction Tools for Targeted Intervention

**DOI:** 10.1371/journal.pone.0167846

**Published:** 2016-12-08

**Authors:** Sara L. White, Debbie A. Lawlor, Annette L. Briley, Keith M. Godfrey, Scott M. Nelson, Eugene Oteng-Ntim, Stephen C. Robson, Naveed Sattar, Paul T. Seed, Matias C. Vieira, Paul Welsh, Melissa Whitworth, Lucilla Poston, Dharmintra Pasupathy, UPBEAT Consortium

**Affiliations:** 1 Division of Women’s Health, King’s College London, London, United Kingdom; 2 MRC Integrative Epidemiology Unit at the University of Bristol, Bristol, United Kingdom; 3 School of Social and Community Medicine, University of Bristol, Bristol, United Kingdom; 4 Guy’s & St Thomas’ NHS Foundation Trust, London, United Kingdom; 5 MRC Lifecourse Epidemiology Unit and NIHR Southampton Biomedical Research Centre, University of Southampton, Southampton, United Kingdom; 6 University Hospital Southampton NHS Foundation Trust, Southampton, United Kingdom; 7 School of Medicine, University of Glasgow, Glasgow, United Kingdom; 8 Institute of Cellular Medicine, Uterine Cell Signalling Group, Newcastle University, Newcastle Upon Tyne, United Kingdom; 9 Institute of Cardiovascular and Medical Sciences, University of Glasgow, Glasgow, United Kingdom; 10 Maternity Services, Central Manchester University Hospitals NHS Foundation Trust, Manchester, United Kingdom; 11 Maternal and Fetal Health Research Centre, University of Manchester, Manchester, United Kingdom; Garvan Institute of Medical Research, AUSTRALIA

## Abstract

All obese women are categorised as being of equally high risk of gestational diabetes (GDM) whereas the majority do not develop the disorder. Lifestyle and pharmacological interventions in unselected obese pregnant women have been unsuccessful in preventing GDM. Our aim was to develop a prediction tool for early identification of obese women at high risk of GDM to facilitate targeted interventions in those most likely to benefit. Clinical and anthropometric data and non-fasting blood samples were obtained at 15^+0^–18^+6^ weeks’ gestation in 1303 obese pregnant women from UPBEAT, a randomised controlled trial of a behavioural intervention. Twenty one candidate biomarkers associated with insulin resistance, and a targeted nuclear magnetic resonance (NMR) metabolome were measured. Prediction models were constructed using stepwise logistic regression. Twenty six percent of women (n = 337) developed GDM (International Association of Diabetes and Pregnancy Study Groups criteria). A model based on clinical and anthropometric variables (age, previous GDM, family history of type 2 diabetes, systolic blood pressure, sum of skinfold thicknesses, waist:height and neck:thigh ratios) provided an area under the curve of 0.71 (95%CI 0.68–0.74). This increased to 0.77 (95%CI 0.73–0.80) with addition of candidate biomarkers (random glucose, haemoglobin A1c (HbA1c), fructosamine, adiponectin, sex hormone binding globulin, triglycerides), but was not improved by addition of NMR metabolites (0.77; 95%CI 0.74–0.81). Clinically translatable models for GDM prediction including readily measurable variables e.g. mid-arm circumference, age, systolic blood pressure, HbA1c and adiponectin are described. Using a ≥35% risk threshold, all models identified a group of high risk obese women of whom approximately 50% (positive predictive value) later developed GDM, with a negative predictive value of 80%. Tools for early pregnancy identification of obese women at risk of GDM are described which could enable targeted interventions for GDM prevention in women who will benefit the most.

## Introduction

Recent estimates suggest that 7 million women were obese in the UK in 2014, and that by 2025, 1 in 5 women in the world will be similarly affected. [1] Obesity is a major risk factor for gestational diabetes (GDM), increasing the likelihood of the disorder 3–5 fold. [2] Women with GDM require intensive antenatal care to achieve optimal blood glucose control and to identify other common obstetric complications, particularly fetal macrosomia and large for gestational age (LGA) infants. [3]

The recent demonstration in a nulliparous prospective cohort of more than 4000 women that diagnosis of GDM is preceded by excessive fetal growth occurring between 20–28 weeks’ gestation, and that this is compounded by maternal obesity, provides a clear rationale for early pregnancy risk identification and intervention to prevent GDM and associated fetal growth. [4] The identification of insulin resistance in the absence of overt diabetes in early pregnancy in obese women provides further reason for targeting treatment to obese women early in gestation [5]. This recognition has led to several recent randomised controlled trials (RCTs) of early interventions in unselected obese women to prevent GDM, including dietary and physical activity advice and pharmacological (metformin) approaches, but the majority have been unsuccessful. [6–9] At present all obese pregnant women are considered to be equally at high risk of developing GDM, whereas approximately only 15–30% (depending on criteria for diagnosis) will develop the disorder. [2] Prediction tools as a means to stratify disease risk are increasingly used in medical [10] and obstetric practice [11] with a focus on precision prevention and treatment for at risk sub-groups. Correctly identifying obese women with heightened risk of GDM early in pregnancy would enable targeted intervention in women most likely to benefit.

There is no accepted strategy to identify obese women at high risk of GDM early in pregnancy. Current clinical risk assessment, such as that recommended by UK National Institute for Health and Care Excellence guidelines, to determine which women should have an oral glucose tolerance test (OGTT) later in pregnancy includes obesity as a risk factor (≥30kg/m^2^). This screening criterion is clearly not applicable for assessment of risk amongst obese women. Previously reported early pregnancy prediction tools for GDM, as yet not adopted in clinical practice, have been constructed in populations unselected for body mass index (BMI). [12–18] With the inclusion of weight or BMI in all tools, performance amongst obese women is likely to be limited.

We have previously established proof of principle for a prediction algorithm combining clinical variables and biomarkers from 106 obese pregnant women who were recruited to the pilot study of UPBEAT, an RCT of a behavioural (diet and physical activity) intervention. [19] The aim of the present study was to develop a simple, robust and easily accessible GDM prediction tool designed specifically for obese women using the entire UPBEAT cohort, with the intention of facilitating early intervention in those women at the highest risk of the disorder. To achieve this aim we measured 21 biomarkers of biological relevance to GDM and a targeted metabolome of 158 metabolites in early pregnancy samples from 1303 women who were obese. Using statistical modelling to combine clinical variables and the best performing biomarkers we developed several prediction tools with potential for early pregnancy stratification for GDM risk.

## Materials and Methods

### Study design

This was a prospective cohort study using clinical data and samples from the UPBEAT trial (ISRCTN 89971375), a multi-centre RCT of a complex dietary and physical activity intervention designed primarily to prevent GDM in obese women, and LGA in their offspring. [8] All participants, including women aged 16 and 17 years (assessed as competent applying Fraser guidelines), provided informed written consent prior to taking part. This process together with all other aspects of the study was approved by the NHS Research Ethics Committee (UK Integrated Research Application System; reference 09/H0802/5). In brief, the UPBEAT cohort comprised 1555 women recruited between 2009 and 2014. Women >16 years of age with a BMI of ≥30kg/m^2^ and a singleton pregnancy were randomised between 15^+0^ and 18^+6^ weeks’ gestation (trial entry) to either standard antenatal care or a physical activity and dietary behavioural intervention superimposed on standard antenatal care. [8, 20] For the purposes of this analysis the trial was treated as a cohort study as the primary outcomes (GDM and LGA infants) did not differ between control and intervention groups. [8]

### Participants

Participants were women recruited to the UPBEAT trial with available OGTT data. The trial protocol stated that an OGTT would be performed between 27^+0^ and 28^+6^ weeks’. We adopted a clinically pragmatic approach and included all OGTTs in a wider time frame (23+0–32^+6^ weeks’; mean 27^+5^). Two individuals were excluded; one because of a positive early OGTT (13^+5^ weeks’), and the second because of an uninterpretable OGTT result.

### Procedures

At trial entry (mean 17^+0^ weeks’) clinical data including socio-demographic and clinical characteristics, medical and family history, and information about the index pregnancy were recorded and non-fasting blood samples taken. Blood (whole blood, plasma and serum) was kept on ice, processed within 2 hours and stored at -80°C. The diagnosis of GDM was according to IADPSG (International Association of Diabetes and Pregnancy Study Groups) criteria, with one or more positive plasma glucose values; fasting ≥5.1 mmol/l, 1 hour ≥10.0 mmol/l, 2 hour ≥8.5 mmol/l, following a 75g oral glucose load. [21]

Three sets of analyses contributed to development of the prediction tools; Model 1—clinical and demographic variables (clinical tool), Model 2—the clinical tool with addition of candidate biomarkers, and Model 3—addition of a targeted nuclear magnetic resonance (NMR) metabolome to Model 2. The rationale was to develop the ‘simplest’ accurate tool. The selection of clinical variables was based on *a-priori* knowledge of plausible association with GDM including age, ethnicity, socioeconomic status (Index of Multiple Deprivation [8]), parity, BMI, previous GDM, family history (first degree relative with hypertension, ischaemic heart disease, GDM or type 2 diabetes mellitus), polycystic ovarian syndrome (self-reported) and smoking (at trial entry). Maternal anthropometric data and blood pressure (BP) measurements were undertaken by staff trained in these measurements. Maternal skinfold thicknesses (triceps, biceps, suprailiac and subscapular) were measured in triplicate, using Harpenden skinfold Calipers (Holtain Ltd, Felin-y-Gigfran, Crosswell, UK). [22] The mean of the three measurements was used for analyses. Maternal circumferences (waist, hip, thigh, neck, mid-arm and wrist) were measured using a calibrated plastic tape; waist—midway between iliac crest and inferior margin of lowest rib; hip—maximum diameter over buttocks; thigh—maximum diameter; neck—midway between mid-cervical spine and mid-anterior neck; mid-arm—diameter midway between elbow and edge of the acromion with arm held straight; wrist—narrowest point around wrist inferior to radial promontory. BP was recorded using the pregnancy validated Microlife BP3BT0-A blood pressure monitor (Microlife, Widnau, Switzerland).

The candidate biomarkers included 21 analytes with *a-priori* associations with insulin resistance, GDM or type 2 diabetes mellitus [23]: adipokines (adiponectin and leptin); inflammatory and endothelial markers (interleukin-6, high sensitivity C-reactive protein and tissue plasminogen activator antigen); lipids (triglycerides, total cholesterol, LDL cholesterol and HDL cholesterol); liver associated markers (aspartate aminotransferase, alanine aminotransferase, gamma-glutamyl transferase, sex hormone binding globulin (SHBG), and ferritin); markers of glucose homeostasis (glucose, insulin, haemoglobin A1c (HbA1c), C-peptide, and fructosamine) and other (vitamin D and human placental lactogen). Analytical methodologies are reported in S1 Table. One hundred and fifty eight metabolites were also measured in serum using an NMR targeted metabolome platform (Brainshake Ltd, http://brainshake.fi/) including 138 lipid measures (lipoprotein particle subclasses, particle size, cholesterols, fatty acids, apolipoproteins, glycerides and phospholipids), and 20 low-molecular weight metabolites including branched chain and aromatic amino acids, glycolysis metabolites, and ketone bodies. A full list of metabolites is presented in S2 Table. This high-throughput NMR metabolomics approach has been widely used in epidemiological studies, [24–27] and experimental details (sample preparation and analysis) have been previously described. [27] All blood samples were processed by laboratory technicians blinded to participant data.

### Statistical analysis

Statistical analysis was performed using Stata software, version 14.0 (StataCorp LP, College Station, Texas). Distributions of all potential predictors were checked for normality. As women were recruited between 15^+0^–18^+6^ weeks’ gestation, appropriate clinical factors and all biochemical variables were checked for variation and transformed into gestational age corrected centiles where required (xriml command, Stata [28]). Summary statistics between those who developed GDM and those who did not were compared using either Student’s *t* test or Mann Whitney tests for continuous data as appropriate and chi-squared tests for categorical data. Candidate biomarkers with a non-parametric distribution were log transformed (base2). Following these transformations regression model assumptions (linear associations) were checked. HDL cholesterol showed a non-linear association and was transformed into a categorical variable using a clinically meaningful threshold. [29]

Three prediction models were developed. Univariate logistic regression was performed on all factors and a pre-defined p value threshold of 0.1 was used to identify predictors for testing in the multivariate models. Clinical variables below the p value threshold were utilised to construct Model 1. Next, the candidate biomarkers identified in univariate regression were incorporated with the selected clinical variables of Model 1, creating Model 2. Finally, selected clinical and candidate biomarker variables from Model 2 were ‘offered’ alongside all identified NMR metabolites to generate Model 3. Forward stepwise logistic regression was used for the development of these models.

Predictive accuracy of the three models was assessed (and compared between models) using the Area Under the Receiver Operator Characteristics Curve (AUC). Model calibration was assessed by discrimination of actual versus predicted GDM risk at differing levels of predicted risk. The test performances of the models were assessed using sensitivity, specificity and positive and negative predictive values at different risk thresholds.

### Translational prediction models for clinical use

In addition to Models 1–3, we explored a range of clinically translatable models (Models 4–8). The variables included were selected from Models 1–3 or correlated measures. Selection was on the basis of established laboratory assays and ease of measurement in the clinic. No stepwise procedures were undertaken for these models.

### Missing data

Missing data for clinical variables was minimal (<1.5%) except for family history of GDM (4%). Candidate biomarkers were available in 73% (n = 953) and NMR metabolites in 69% (n = 895). Most missing blood biomarker data was because participants did not provide a blood sample. All models were constructed using complete data based on each group of factors (clinical, candidate biomarker and metabolome). The sample for Model 1 (clinical model) and Model 3 (including candidate biomarker and metabolome) were 1267 and 770 respectively. All other models used a single data set with complete data for the main clinical and candidate biomarkers (Model 2, 4–8, n = 805). We explored the possibility of bias due to missing data by comparing associations of clinical predictors with GDM in the sample with maximal data (Model 1, clinical factors) to the same associations assessed in the complete case samples (Model 2 and Model 3, biomarkers and metabolome respectively).

### Validation of the prediction model

Two methods of ten-fold cross validation were used for internal validation of the different models. [30, 31]

### Sensitivity analyses

As women with a previous history of GDM are frequently considered as a high risk sub-group necessitating specific management, [3] multivariable and discrimination analyses were repeated for Models 1–3 following removal of women with previous GDM (n = 25).

## Results

**Fig 1:**
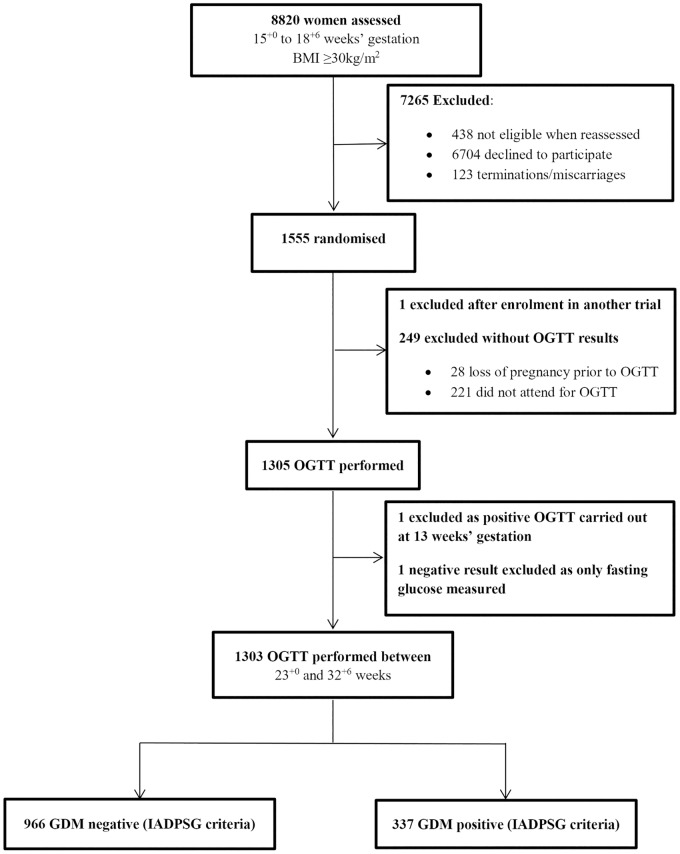
Study population.

**Fig 2:**
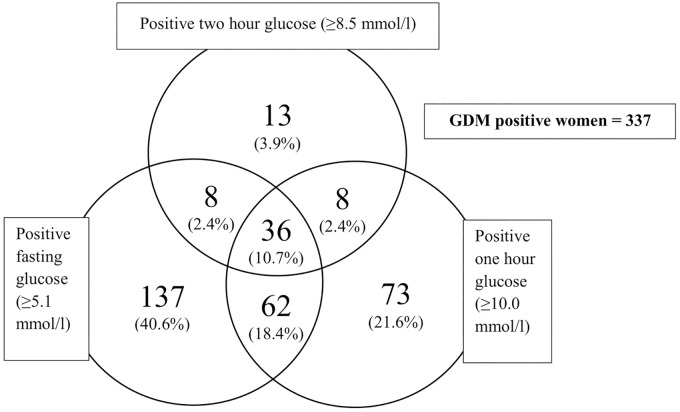
Spread of time points for positive glucose results leading to GDM diagnosis.

Of the 1555 participants in the UPBEAT trial, 1303 were included in this study (median BMI 35 kg/m^2^). Of these, 337 (25.9%) developed GDM (Fig 1). The diagnosis of GDM in the majority of women was based on elevated fasting glucose (72%). A further 24% and 4% were because of raised 1-hour and 2-hour post-load glucose respectively (Fig 2). Women with GDM were older than women who did not develop GDM, and more likely to have had GDM in a previous pregnancy or a first-degree relative with type 2 diabetes mellitus. BMI and BP (systolic and diastolic) were higher in those with GDM, as were skinfold thicknesses and neck, waist, hip, wrist and mid-arm circumferences (Table 1). In univariate analysis, most of the candidate biomarkers and many of the NMR metabolites were associated with GDM (Table 2; S3 Table). GDM related NMR metabolites included lipoprotein particle subclasses, some fatty acids, amino acids and ketone bodies.

**Table 1:** Maternal characteristics by GDM status.

	No GDM (n = 966) Mean (SD) or N (%)	GDM (n = 337) Mean (SD) or N (%)	p-value ^a^
**Age (years)**	30.3 (5.5)	32.0 (4.9)	<0.001
**Ethnicity**			
African	151 (15.6)	64 (19.0)	0.60
African Caribbean	74 (7.6)	22 (6.5)	
South Asian	55 (5.7)	21 (6.2)	
European	616 (63.8)	204 (60.5)	
Other	70 (7.2)	26 (7.7)	
**Adjusted English & Scottish IMD** ^b^ ^c^			
least deprived	229 (23.8)	69 (20.5)	0.31
Intermediate	339 (35.3)	115 (34.2)	
most deprived	393 (40.9)	152 (45.2)	
**Parity**			
Nulliparous	435 (45.0)	143 (42.4)	0.62
**Previous GDM**	11 (1.1)	14 (4.2)	0.002
**PCOS** ^b^	85 (8.9)	38 (11.4)	0.18
**Current smoking**	60 (6.2)	28 (8.3)	0.19
**Family history**			
T2DM ^b^	204 (21.2)	104 (30.9)	<0.001
GDM ^b^	34 (3.6)	17 (5.2)	0.20
IHD ^b^	140 (14.5)	58 (17.2)	0.24
HTN ^b^	430 (44.6)	163 (48.4)	0.23
**Pregnancy outcome**			
Pre-eclampsia ^b^	32 (3.4)	18 (5.5)	0.092
Postpartum haemorrhage (≥1000ml) ^b^	127 (13.4)	53 (16.1)	0.215
Caesarean section (all) ^b^	331 (34.4)	139 (41.6)	0.019
Caesarean section (emergency) ^b^	164 (17.1)	58 (17.4)	0.9
Gestational age at delivery (weeks) ^b^ ^d^	40.1 (39.1–41.1)	38.7 (38.1–39.9)	<0.001
Preterm delivery (<37 weeks’) ^b^	42 (4.4)	22 (6.6)	0.1
Birthweight (g) ^b^	3457 (567)	3396 (537)	0.09
LGA (≥90^th^ customised centile) ^b^	66 (6.9)	42 (12.6)	0.001
NICU admission ^b^	64 (6.7)	34 (10.2)	0.036
**Clinical examination**			
BMI (kg/m^2^) ^d^	34.7 (32.7–38.1)	36.2 (33.1–39.9)	<0.001
Systolic BP (mmHg) ^b^	116.7 (10.8)	120.9 (10.9)	<0.001
Diastolic BP (mmHg) ^b^	71.4 (7.6)	74.3 (8.0)	<0.001
**Skinfolds (mean, mm)** ^b^			
Triceps	32.5 (8.7)	34.7 (9.6)	<0.001
Biceps ^d^	20.3 (16–25)	21.8 (17–28)	<0.001
Subscapular	34.4 (9.5)	38.3 (10.8)	<0.001
Suprailiac	31.3 (10.9)	34.7 (11.1)	<0.001
Sum of skinfolds	119.5 (25.7)	131.2 (29.3)	<0.001
Neck circumference (cm) ^b^	36.3 (2.4)	37.4 (2.5)	<0.001
Waist circumference (cm) ^b^ ^d^	105 (99–112)	110 (103–116)	<0.001
Mid-arm circumference (cm) ^b^ ^d^	36 (34–38)	37 (35–40)	<0.001
Wrist circumference (mm)^b^ ^d^	170 (161–180)	172 (165–180)	0.02
Hip circumference (cm)^b^ ^d^	121 (116–127)	123 (116–130)	0.04
Thigh circumference (cm) ^b^	68.4 (6.3)	68.9 (7.6)	0.24
Waist:hip ratio ^b^	0.87 (0.08)	0.89 (0.07)	<0.001
Waist:thigh ratio^b^ ^d^	1.54 (1.43–1.66)	1.61 (1.5–1.71)	<0.001
Neck:thigh ratio ^b^	0.53 (0.05)	0.55 (0.07)	<0.001
Waist:height ratio^b^ ^d^	0.64 (0.60–0.68)	0.66 (0.63–0.72)	< 0.001

GDM—gestational diabetes, IMD—index of multiple deprivation, PCOS—polycystic ovarian syndrome, T2DM—type 2 diabetes mellitus, IHD—ischaemic heart disease, HTN—hypertension, LGA—large for gestational age, NICU—neonatal intensive care unit, BMI—body mass index

^a^ p-value from Student’s *t* test, Mann Whitney test or chi-squared test.

^b^ Missing data at baseline: IMD (n = 6), PCOS (n = 10), 1^st^ degree relative T2DM (n = 2), 1^st^ degree relative GDM (n = 53), 1^st^ degree relative IHD (n = 2), 1^st^ degree relative HTN (n = 2), LGA (n = 8), pre-eclampsia (n = 27), postpartum haemorrhage (n = 23), caesarean section (n = 8), gestational age at delivery (n = 8), preterm birth (n = 8), birthweight (n = 8), systolic and diastolic BP (n = 16), skinfolds—triceps (n = 8), biceps (n = 10), subscapular (n = 9), suprailiac (n = 10), sum of skinfolds (n = 13), circumferences—neck (n = 6), waist (n = 6), mid-arm (n = 7), wrist (n = 11), hip (n = 6), thigh (n = 6), ratios (n = 6).

^c^ IMD categories: least deprived represent 1^st^, 2^nd^ and 3^rd^ quintiles of IMD distribution. Intermediate represents 4^th^ quintile, most deprived represents women in 5^th^ quintile.

^d^ Mann Whitney test (median, IQR)

**Table 2:** Biomarkers measured at trial entry by GDM status.

Biomarker ^a^	No GDM (n = 678) Mean (SD)	GDM (n = 275) Mean (SD)	p-value ^b^
t-PA antigen (ng/ml) ^c^	2.7 (0.8)	2.8 (0.8)	0.07
Total cholesterol (mmol/l)	5.7 (1.0)	5.7 (1.0)	0.81
LDL cholesterol (mmol/l)	2.8 (0.8)	2.8 (0.8)	0.48
Glucose (mmol/l)	4.7 (0.7)	5.1 (0.9)	<0.001
Fructosamine (umol/l)	185.1 (20.3)	190.9 (22.5)	<0.001
SHBG (nmol/l)	437.2 (127.8)	386.7 (109.9)	<0.001
HbA1c (%)	4.8 (0.3)	5.0 (0.4)	<0.001
HbA1c (mmol/mol)	28.9 (3.6)	30.9 (4.1)	<0.001
Insulin (mU/l) ^c^	4.6 (1.4)	5.1 (1.3)	<0.001
C-peptide (ng/ml) ^c^	1.9 (0.8)	2.2 (0.8)	<0.001
hs-CRP (mg/L) ^c^	2.6 (1.1)	2.8 (1.1)	0.01
gGT (U/L) ^c^	3.6 (1.0)	4.0 (1.0)	<0.001
ALT (U/L) ^c^	4.1 (0.8)	4.2 (0.7)	0.32
AST (U/L) ^c^	4.5 (0.5)	4.5 (0.5)	0.10
Triglycerides (mmol/L) ^c^	0.7 (0.5)	0.9 (0.5)	<0.001
Leptin (pg/ml) ^c^	6.0 (0.6)	6.1 (0.7)	0.04
Adiponectin (ug/ml) ^c^	3.5 (0.9)	3.0 (0.9)	<0.001
Ferritin (ng/ml) ^c^	5.5 (1.1)	5.7 (1.0)	0.05
IL-6 (pg/ml) ^c^	1.6 (0.9)	1.7 (0.9)	0.20
Vitamin D (ng/ml) ^c^	3.9 (0.9)	3.8 (0.9)	0.04
Human placental lactogen (z score)	0.1 (1.0)	-0.2 (1.0)	<0.001
HDL cholesterol (mmol/l)	**Number** (%)	**Number** (%)	
>1.5	344 (51.3)	112 (40.9)	0.003

GDM—gestational diabetes, t-PA antigen—tissue plasminogen activator antigen, LDL—low density lipoprotein, SHBG—sex hormone binding globulin, HbA1c –haemoglobin A1c, hs-CRP—high sensitivity C-reactive protein, gGT—gamma-glutamyl transferase, ALT—alanine aminotransferase, AST—aspartate aminotransferase, IL-6 –interleukin-6, hPL—human placental lactogen, HDL—high density lipoprotein.

^a^ all biomarkers had missing values: tPA antigen (n = 11), total cholesterol (n = 6), LDL and HDL cholesterol (n = 9), glucose (n = 20), fructosamine (n = 13), SHBG (n = 18), HbA1c (n = 75), insulin (n = 6), C-peptide (n = 17), hs-CRP (n = 9), gGT (n = 9), ALT (n = 7), AST (n = 8), triglycerides (n = 9), leptin (n = 12), adiponectin (n = 12), ferritin (n = 13), IL-6 (n = 11), vitamin D (n = 22), hPL (n = 30), HDL cholesterol (n = 9).

^b^ p-value from Student’s *t* test or chi-squared test.

^c^ Transformed to log base 2.

Models 1 (clinical factors), Model 2 (plus candidate biomarkers) and Model 3 (Model 2 plus metabolome) are shown in Table 3. A clinical tool including previous GDM, age, systolic BP, sum of maternal skinfold thicknesses and anthropometric ratios (waist:height and neck:thigh) showed good discrimination (AUC 0.71, 95% CI 0.68–0.74). This improved with addition of candidate biomarkers to 0.77 (95% CI 0.73–0.80) (p <0.001 *vs* Model 1). Candidate biomarkers contributing to this model were HbA1c, glucose, fructosamine, triglycerides, adiponectin and SHBG. The contribution of some clinical factors selected in Model 1 was attenuated by addition of these biomarkers. The addition of the NMR metabolites (Model 3) did not improve upon the performance of Model 2 (AUC 0.77, 95% CI 0.74–0.81; p = 0.22 *vs* Model 2). All three models were well calibrated (S4 Table).

**Table 3:** GDM prediction models.

	Model 1 (n = 1267) OR (95% CI)	Model 2 (n = 805) OR (95% CI)	Model 3 (n = 770) OR (95% CI)	Model 4 (n = 805) OR (95% CI)	Model 5 (n = 805) OR (95% CI)	Model 6 (n = 805) OR (95% CI)	Model 7 (n = 805) OR (95% CI)	Model 8 (n = 805) OR (95% CI)
**No of cases of GDM (%)**	329 (25.9)	241 (29.9)	232 (30.1)	241 (29.9)	241 (29.9)	241 (29.9)	241 (29.9)	241 (29.9)
**Clinical**								
Age (years)	1.06 (1.03–1.09)	1.05 (1.01–1.08)	1.04 (1.00–1.08)	1.05 (1.02–1.08)	1.05 (1.02–1.08)	1.05 (1.02–1.08)	1.05 (1.02–1.09)	
Previous GDM ^a^	3.47 (1.45–8.30)							
1st degree relative T2DM	1.40 (1.03–1.89)							
Sum of skinfold thicknesses (mm)	1.01 (1.00–1.02)	1.01 (1.01–1.02)	1.01 (1.01–1.02)					
Waist:height ratio (per 0.1)	1.57 (1.25–1.98)							
Neck:thigh ratio (per 0.1)	1.55 (1.23–1.95)	1.52 (1.11–2.06)	1.52 (1.11–2.08)					
Systolic BP (per 10 mmHg)	1.36 (1.20–1.54)	1.23 (1.06–1.44)	1.22 (1.04–1.43)	1.25 (1.07–1.45)	1.24 (1.07–1.44)	1.23 (1.06–1.43)	1.27 (1.10–1.48)	
Mid-arm circumference (cm)				1.03 (0.99–1.08)			1.03 (0.99–1.07)	
Subscapular skinfold thickness (mm)					1.03 (1.01–1.04)			
Waist circumference (cm)						1.02 (1.01–1.04)		
**Candidate biomarkers**								
HbA1C (mmol/mol)		1.11 (1.06–1.16)	1.10 (1.05–1.16)	1.11 (1.06–1.16)	1.11 (1.06–1.16)	1.11 (1.06–1.16)	1.11 (1.06–1.16)	1.12 (1.08–1.17)
Random glucose (mmol/l)		1.52 (1.22–1.89)	1.63 (1.30–2.06)	1.66 (1.35–2.05)	1.65 (1.33–2.04)	1.66 (1.34–2.05)		
Fructosamine (per 10umol/l)		1.11 (1.02–1.20)	1.13 (1.04–1.23)					
SHBG (per 10nmol/l)		0.97 (0.96–0.99)	0.98 (0.96–0.99)					
Adiponectin (ug/ml) ^b^		0.73 (0.60–0.89)	0.73 (0.60–0.90)	0.60 (0.50–0.73)	0.62 (0.52–0.75)	0.61 (0.51–0.74)	0.58 (0.48–0.70)	0.59 (0.49–0.70)
Triglycerides (mmol/l) ^b^		1.60 (1.08–2.38)						
**Metabolome**								
Triglycerides in medium HDL (per 10umol/l)			1.36 (1.14–1.61)					
3-Hydroxybutyrate (per 10umol/l)			1.03 (1.01–1.06)					
**AUC**	0.71 (0.68–0.74)	0.77 (0.73–0.80)	0.77 (0.74–0.81)	0.72 (0.68–0.76)	0.73 (0.69–0.77)	0.73 (0.69–0.77)	0.71 (0.67–0.75)	0.68 (0.64–0.72)
Internal validation: standard method	0.70 (0.66–0.74)	0.75 (0.70–0.81)	0.75 (0.69–0.81)	0.71 (0.67–0.76)	0.72 (0.67–0.77)	0.72 (0.67–0.77)	0.71 (0.67–0.75)	0.68 (0.63–0.73)
Altman method ^c^	0.69 (0.65–0.72)	0.73 (0.68–0.78)	0.73 (0.66–0.80)					

GDM—gestational diabetes, OR—odds ratio, T2DM—type 2 diabetes mellitus, HbA1c –haemoglobin A1c, HDL—high density lipoprotein

Model 1: Clinical model only

Model 2: Clinical plus candidate biomarker model

Model 3: Clinical plus candidate biomarker plus metabolome model

Model 4–8: Clinically translatable models

^a^ The referent category for this predictor was multiparous women without previous history of GDM. Nulliparous obese women were not at increased risk of GDM OR 1.17 (95% CI 0.88–1.55) when compared to this referent group.

^b^ log transformed to base2

^c^ Altman method was not used for internal validation in models 4–8 as stepwise procedures were not used for selection of factors in these models.

Models that would easily translate to the clinical setting were also explored. These focused on readily attainable clinical variables, and biomarkers with established and inexpensive assays. Models showed good levels of discrimination (AUC >0.70) (Table 3). Models also showed a high level of internal validity (Table 3).

Sensitivity, specificity and positive and negative predictive values were estimated at different risk thresholds for all models. Different thresholds were explored to balance sensitivity and specificity. Setting the estimated risk for GDM at ≥35% as identifying the high-risk sub-group, approximately 50% of this group progressed to GDM, with 80% of those not developing GDM correctly identified as not at risk (Table 4).

**Table 4:** Performance of models predicting GDM at risk threshold of ≥35%.

Model	Sensitivity	Specificity	PPV	NPV
Model 1	41.0	83.8	47.0	80.2
Model 2	59.8	78.5	54.3	82.0
Model 3	58.6	78.1	53.5	81.4
Model 4	54.8	77.7	51.2	80.1
Model 5	56.0	78.9	53.1	80.8
Model 6	56.4	77.3	51.5	80.6
Model 7	52.7	75.7	48.1	78.9
Model 8	47.3	76.1	45.8	77.2

GDM—gestational diabetes, PPV—positive predictive value, NPV—negative predictive value

Model 1: Clinical model only

Model 2: Clinical plus candidate biomarker model

Model 3: Clinical plus candidate biomarker plus metabolome model

Model 4–8: Clinically translatable models

When compared to the analyses in the maximal number (n = 1267), the selected variables, their magnitude of associations and the AUC were similar in the sub-groups that were included in Model 2 (n = 805) (S5 Table). In the sub-group used in Model 3 (n = 770) the magnitudes of associations of anthropometric predictors with GDM were stronger and an additional predictor (waist:thigh) appeared in the clinical model for this sub-sample (S5 Table). In both these sub-samples previous history of GDM did not appear as a predictor despite its strong magnitude of association in the larger sample of 1267 women. This is likely related to the smaller numbers of women with previous history of GDM in models 2 and 3 (n = 14, and n = 13 respectively). Sensitivity analysis removing women with a previous history of GDM from Models 1–3 identified similar clinical predictors, candidate biomarkers and NMR metabolites. These models included an additional anthropometric measure (waist:thigh), strengthened the association of previously identified anthropometric measures, and identified further candidate biomarkers and metabolites from the metabolome (S6 Table). The AUC were similar to those found in the primary analysis in Models 1–3.

## Discussion

Current clinical guidelines for GDM risk assessment do not differentiate between obese women with differing metabolic risk. In this, the largest and most comprehensive study to date to investigate early pregnancy risk factors for later onset of GDM in obese women, we have used an extensive range of clinical and biomarker variables to develop prediction tools to identify obese women at high risk of GDM. The model with best discrimination combined four clinical characteristics with six candidate biomarkers. Models that focused on a few clinical factors and biomarkers readily available in clinical practice, and with minimal cost, also performed well. In addition, we identified a model that does not require blood sampling, which could be developed for low and middle income countries where the prevalence of GDM and obesity is rapidly increasing. [32] Clinical use of tests such as these which provide risk assessment at the first antenatal visit would enable prompt intervention (behavioural or pharmacological) in at risk women. Women identified as being at low risk of developing GDM should be managed according to clinical guidelines for all pregnant obese women, including dietary advice, and be alerted to potential risks during pregnancy and beyond.

The decision to focus on obese pregnant women was predicated by the increasing prevalence of obesity and GDM, the lack of predictive algorithms specific to obese women and because mechanistic pathways leading to GDM may differ in obese compared with normal weight women. Obesity related GDM is initially associated with insulin resistance [33] whereas in lean women an inadequate insulin secretory response to the physiological state of insulin resistance in pregnancy is considered predominant. [34] The recognition that the metabolic defects of maternal obesity are potentially modifiable has stimulated several well conducted RCTs that have tested behavioural (dietary and/or physical activity changes) and a pharmacological intervention (metformin) in early pregnancy to prevent GDM and associated adverse pregnancy outcomes. [6–9] Most have been ineffective, which we hypothesise is because treatment is more likely to benefit only the sub-group with the highest metabolic risk. Having demonstrated the ability to better identify obese women at risk of GDM there is now the potential through new RCTs to address this hypothesis.

As guidelines frequently recommend specific care pathways for women with a previous history of GDM we performed sensitivity analysis excluding these women. Since GDM discrimination remained similar for later GDM across the three comprehensive prediction models (Models 1–3), there was no rationale to support exclusion of previous GDM.

BMI which is recognised, especially in pregnancy, to be a poor index of fat mass, was superseded in the statistical models by other anthropometric measures, three of which were independent predictors of GDM. To our knowledge these simple measures, whilst recognised in a few earlier reports, [35–37] have been largely ignored in assessment of GDM risk. In contrast to clinical guidelines for GDM in the whole population, we found no evidence of a strong role of ethnicity in this mixed ethnic population.

To our knowledge this study is the first to address a wide range of biomarkers in prediction of GDM in obese women. As anticipated, most candidate biomarkers were individually associated with the disorder. The identification of adiponectin in Models 2 and 3 concurs with a recent review which reported that this adipokine is a predictor of GDM in women of mixed BMI. [38] Of the NMR metabolites univariably associated with GDM, 56 were lipoprotein particle subclasses, with the majority being very large density lipoprotein particle subtypes. Branched chain amino acids, previously associated with insulin resistance and type 2 diabetes mellitus, [39, 40] were also associated. As only 2 of these metabolites were selected for inclusion in the combined model, and did not improve the test performance, this metabolome is unlikely to provide valuable biomarkers for clinical GDM prediction.

Current practice for evaluating GDM risk in pregnant women does not include risk stratification amongst obese women, treating all those with a BMI of ≥30kg/m^2^ as high risk. With escalating rates of obesity worldwide, these models will become increasingly unuseful. The clinical risk factors in the simple tools are quick to measure with minimal training, and the biomarkers, HbA1c and adiponectin are readily accessible for routine clinical laboratory measurement. An added advantage is that the samples were non-fasted; there are practical and ethical issues in asking women in early pregnancy to attend a clinic appointment in the fasted state, and this is not current practice in the UK. We acknowledge that a fasting sample may have provided additional predictive potential, however the original study protocol was designed pragmatically to allow simple clinical translatability.

Following a recent report of strong associations between abnormal OGTTs, particularly raised fasting glucose, in obese women in early pregnancy and measures of insulin resistance [5], an alternative to the use of an early prediction tool as described here might be to bring forward the OGTT or simply measure fasting glucose. Other than the practicalities of fasting, an early OGTT has yet to be validated in regard to maternal and neonatal outcomes or GDM, as traditionally diagnosed. Moreover, several studies in BMI heterogeneous populations have suggested that an early abnormal glucose test is not adequately sensitive or specific to replace a later OGTT for the diagnosis of GDM. [41, 42] All such approaches require further validation and randomised controlled trials to determine the efficacy of either lifestyle or pharmacological interventions in early pregnancy targeted to those identified at risk.

### Study strengths and limitations

Strengths include novelty, large sample size and mixed ethnicity of the study population, as well as identification of a range of models with immediate clinical applicability. One limitation in terms of current practice could be the use of IADPSG criteria to define GDM, which although recommended by the World Health Organization [43] is not universally adopted. [3] However, when tested using the current UK diagnostic criteria [3], performance of the prediction tools was comparable. Up to 40% of participants had missing data on one or more of the blood based biomarkers or clinical characteristics and sensitivity analysis did demonstrate some differences in smaller sub-samples, but performance was broadly similar. Because of the uniqueness of the data collection, external validation of the full model was not feasible but validity was strongly supported by internal validation, using two complementary methods. [30, 31] We recognise that the study population may not be representative of a ‘normal’ obstetric population as they were participants in an RCT, an important reason for pursuing external validation of the predictive models. Future evalution studies could also include validation at earlier gestations. Previous GDM did not contribute to Models 2 and 3, but as only 25 women in the cohort had GDM previously, of whom 11 did not develop the disorder in the index pregnancy, a previous history of GDM should be considered for inclusion in future validation studies. Whilst ethnicity was not selected as a predictor in our mixed ethnic population, repetition in specific ethnic groups would be valuable.

In summary, we have demonstrated a method to more accurately identify obese women at high risk of GDM than currently practised. To date, no early pregnancy intervention in obese women has successfully reduced the risk of GDM. The use of the tools described has the potential to enable targeted intervention for those at highest risk and therefore likely to benefit most.

## Supporting Information

S1 Table Candidate biomarker analytical methodologies. (DOCX)

S2 Table Metabolites measured as part of targeted NMR metabolome. (DOCX)

S3 Table Targeted NMR metabolome associated with GDM. (DOCX)

S4 Table Calibration of Models 1–3. (DOCX)

S5 Table Exploring potential impact of missing data by examining clinical predictor associations with GDM on different subsamples. (DOCX)

S6 Table Sensitivity analysis for Models 1–3 excluding women with previous GDM. (DOCX)
