# Supplementary material for: Early Antenatal Prediction of Gestational Diabetes in Obese Women: Development of Prediction Tools for Targeted Intervention
Source: PLoS One. 2016 Dec 8;11(12):e0167846. doi: 10.1371/journal.pone.0167846 (PMC5145208; doi:10.1371/journal.pone.0167846)
Supplement: S1 Table — (DOCX) [file pone.0167846.s001.docx]

S1 Table**. Candidate biomarker analytical methodologies**

| **Biomarker** | **Method** | **Platform** | **CV** |
| --- | --- | --- | --- |
| tPA antigen | Enzyme-linked immunosorbent assay | Asserchrom tpa (Stago) | intra 5.7%, inter 7.9% |
| Total cholesterol | Enzymatic, colorimetric | Roche, Cobas c311 | low 1.8%, high 1.1% |
| LDL cholesterol | Homogenous enzymatic, colorimetric | Roche, Cobas c311 | low 2.0% |
| HDL cholesterol | Homogenous enzymatic, colorimetric | Roche, Cobas c311 | low 2.0% |
| Glucose | Enzymatic, hexokinase | Roche, Cobas c311 | low 2.4%, high 1.5% |
| Fructosamine | Colorimetric, nitroblue tetrazolium | Roche, Cobas c311 | low 3.4% |
| SHBG | Electrochemiluminescence immunoassay | Roche, Cobas e411 | low 5.9%, high 7.9% |
| HbA1c | Turbidimetric inhibition immunoassay | Roche, Cobas c311 | low 1.4%, high 1.3% |
| Insulin | Electrochemiluminescence immunoassay | Roche, Cobas e411 | low 7.8%, high 5.4% |
| C peptide | Electrochemiluminescence immunoassay | Roche, Cobas e411 | low 6.2%, high 5.1% |
| hs-CRP | Particle enhanced immunoturbidimetric | Roche, Cobas c311 | low 7.1% |
| gGT | Enzymatic, colorimetric | Roche, Cobas c311 | low 3.9%, high 3.9% |
| ALT | Enzymatic, spectrophotometric | Roche, Cobas c311 | low 3.3%, high 3.1% |
| AST | Enzymatic, spectrophotometric | Roche, Cobas c311 | low 2.1%, high 1.8% |
| Triglycerides | Enzymatic, colorimetric | Roche, Cobas c311 | low 2.2%, high 1.8% |
| Leptin | Enzyme-linked immunosorbent assay | R and D Systems | intra 2.0%, Inter 9.3% |
| Adiponectin | Enzyme-linked immunosorbent assay | R and D Systems | intra 5.4%, inter 12.0% |
| Ferritin | Particle enhanced immunoturbidimetric | Roche, Cobas c311 | low 1.7% |
| IL-6 | Enzyme-linked immunosorbent assay | R and D Systems | intra 9.8%, inter 12.8% |
| hPL | Enzyme-linked immunosorbent assay | R and D Systems | inter and intra <5.0% |
| Vitamin D | Electrochemiluminescence immunoassay | Roche, Cobas e411 | low 11.2%, high 9.2% |

CV – coefficient of variation, t-PA antigen – tissue plasminogen activator antigen, LDL – low density lipoprotein, HDL – high density lipoprotein, HbA1c – haemoglobin A1c, hs-CRP – high sensitivity C-reactive protein, gGT – gamma-glutamyl transferase, ALT – alanine aminotransferase, AST – aspartate aminotransferase, IL-6 – interleukin-6, hPL - human placental lactogen
