# Supplementary material for: Early Antenatal Prediction of Gestational Diabetes in Obese Women: Development of Prediction Tools for Targeted Intervention
Source: PLoS One. 2016 Dec 8;11(12):e0167846. doi: 10.1371/journal.pone.0167846 (PMC5145208; doi:10.1371/journal.pone.0167846)
Supplement: S2 Table — (DOCX) [file pone.0167846.s002.docx]

S2 Table**. Metabolites measured as part of targeted NMR metabolome**

| **Metabolite group** | **Metabolites** |
| --- | --- |
| **Fatty acids** | Total fatty acids, estimated degree of unsaturation |
|  | FAw3, FAw6, PUFA, MUFA, SFA, DHA, LA |
| **Fatty acid ratios** | DHA/FA, LA/FA, CLA/FA, FAw3/FA, FAw6/FA, PUFA/FA, MUFA/FA, SFA/FA |
| **Lipoprotein subclasses** | **Lipid measures for each subclass** |
| 6 VLDL subclasses (extremely large, very large, large, medium, small, very small) | Esterified cholesterol, free cholesterol, triglycerides, phospholipids,  total cholesterol, total lipids, particle concentration |
| IDL |  |
| 3 LDL subclasses (large, medium, small) |  |
| 4 HDL subclasses (very large, large, medium, small) |  |
| **Lipoprotein particle size** | Mean diameter: VLDL particles, LDL particles, HDL particles |
| **Apolipoproteins** | ApoA-I, ApoB, ApoB/ApoA-l |
| **Cholesterol** | Total cholesterol in VLDL, LDL, HDL, HDL2, HDL3 |
|  | Serum total cholesterol, remnant cholesterol, esterified and free cholesterol |
| **Glycerides and Phospholipids** | Serum total triglycerides, triglycerides in VLDL, LDL, HDL, |
|  | Total phosphoglycerides, ratio of triglycerides to phosphoglycerides |
|  | Phosphatidylcholine and other cholines, sphingomyelins, total cholines |
| **Glycolysis related metabolites** | Glucose, lactate, pyruvate, citrate, glycerol |
| **Amino acids** | Alanine, glutamine, glycine, histadine |
| Branched chain amino acids | Isoleucine, leucine, valine |
| Aromatic amino acids | Phenylalaine, tyrosine |
| **Ketone bodes** | Acetate, acetoacetate, 3-hydroxybutyrate |
| **Fluid Balance** | Creatinine, albumin |
| **Inflammation** | Glycoprotein acetlys, mainly alpha-1 glycoprotein |

NMR – nuclear magnetic resonance, FAw3 - omega-3, FAw6 - omega-6, PUFA - polyunsaturated fatty acids, MUFA - monounsaturated fatty acids; 16:1, 18:1, SFA - saturated fatty acids, DHA - docosahexanaenoic acid 22:6, LA - linoleic acid 18:2, VLDL – very low density lipoprotein, IDL – intermediate density lipoprotein, LDL – low density lipoprotein, HDL – high density lipoprotein
