# Supplementary material for: Early Antenatal Prediction of Gestational Diabetes in Obese Women: Development of Prediction Tools for Targeted Intervention
Source: PLoS One. 2016 Dec 8;11(12):e0167846. doi: 10.1371/journal.pone.0167846 (PMC5145208; doi:10.1371/journal.pone.0167846)
Supplement: S3 Table — (DOCX) [file pone.0167846.s003.docx]

S3 Table**. Targeted NMR metabolome associated with GDM.**

| **Metabolite group** | **Metabolite** | **Units** | **No GDM**  **(n=633)**  **Mean (SD)** | **GDM**  **(n=262)**  **Mean (SD**) | **p-value *** |
| --- | --- | --- | --- | --- | --- |
| **Lipoprotein subclass** | Concentration of chylomicrons and extremely large VLDL particles † | pmol/l | 55.7 (24.8-104.1) | 74.6 (35.4-133.7) | <0.001 |
|  | Total lipids in chylomicrons and extremely large VLDL † | umol/l | 12.6 (5.74-22.8)) | 16.4 (8.10-29.2) | <0.001 |
|  | Phospholipids in chylomicrons and extremely large VLDL † | umol/l | 1.62 (0.71-2.97) | 2.31 (1.06-3.83) | <0.001 |
|  | Total cholesterol in chylomicrons and extremely large VLDL † | umol/l | 3.26 (1.76-5.09) | 4.02 (2.14-6.52) | <0.001 |
|  | Cholesterol esters in chylomicrons and extremely large VLDL † | umol/l | 1.91 (1.10-3.07) | 2.41 (1.39-3.67) | <0.001 |
|  | Free cholesterol in chylomicrons and extremely large VLDL † | umol/l | 1.24 (0.63-2.14) | 1.66 (0.87-2.72) | <0.001 |
|  | Triglycerides in chylomicrons and extremely large VLDL † | umol/l | 7.71 (3.02-14.6) | 10.3 (4.55-18.6) | <0.001 |
|  | Concentration of very large VLDL particles † | nmol/l | 0.46 (0.25-0.76) | 0.59 (0.33-0.98) | <0.001 |
|  | Total lipids in very large VLDL † | umol/l | 45.1 (24.6-74.4) | 57.1 (32.0-96.5) | <0.001 |
|  | Phospholipids in very large VLDL † | umol/l | 7.02 (3.79-12.0) | 9.39 (4.88-16.1) | <0.001 |
|  | Total cholesterol in very large VLDL † | umol/l | 9.45 (5.2-15.0) | 12.2 (6.49-19.7) | <0.001 |
|  | Cholesterol esters in very large VLDL † | umol/l | 5.51 (3.10-8.44) | 6.89 (3.67-10.7) | <0.001 |
|  | Free cholesterol in very large VLDL † | umol/l | 3.94 (2.14-6.67) | 5.19 (2.75-8.81) | <0.001 |
|  | Triglycerides in very large VLDL † | umol/l | 28.4 (15.1-46.5) | 36.6 (20.4-61.0) | <0.001 |
|  | Concentration of large VLDL particles † | nmol/l | 3.49 (2.22-5.24) | 4.29 (2.77-6.42) | <0.001 |
|  | Total lipids in large VLDL † | umol/l | 200.2 (127.6-303) | 247.5 (158.8-372) | <0.001 |
|  | Phospholipids in large VLDL † | umol/l | 37.0 (23.5-55.8) | 45.4 (28.8-67.5) | <0.001 |
|  | Total cholesterol in large VLDL † | umol/l | 45.7 (28.0-68.5) | 56.2 (33.9-84.1) | <0.001 |
|  | Cholesterol esters in large VLDL † | umol/l | 25.3 (16.4-36.8) | 30.3 (19.4-43.9) | 0.0001 |
|  | Free cholesterol in large VLDL † | umol/l | 20.7 (12.0-32.3) | 26.1 (15.2-40.1) | <0.001 |
|  | Triglycerides in large VLDL † | umol/l | 119.1 (74.9-179.1) | 144.7 (95.9-218.9) | <0.001 |
|  | Concentration of medium VLDL particles | nmol/l | 14.7 (6.5) | 16.8 (7.3) | <0.001 |
|  | Total lipids in medium VLDL | umol/l | 493.9 (215.8) | 563.1 (242.0) | <0.001 |
|  | Phospholipids in medium VLDL | umol/l | 100.3 (42.0) | 113.8 (46.9) | <0.001 |
|  | Total cholesterol in medium VLDL | umol/l | 143.1 (55.8) | 159.5 (60.3) | <0.001 |
|  | Free cholesterol in medium VLDL | umol/l | 58.9 (27.6) | 68.2 (31.0) | <0.001 |
|  | Triglycerides in medium VLDL | umol/l | 250.4 (120.5) | 289.8 (137.7) | <0.001 |
|  | Concentration of small VLDL particles | nmol/l | 28.3 (8.1) | 30.6 (8.5) | <0.001 |
|  | Total lipids in small VLDL | umol/l | 564.1 (155.9) | 607.4 (163.6) | <0.001 |
|  | Phospholipids in small VLDL | umol/l | 134.8 (33.7) | 144.4 (35.6) | <0.001 |
|  | Free cholesterol in small VLDL | umol/l | 85.0 (22.1) | 91.2 (23.2) | <0.001 |
|  | Triglycerides in small VLDL | umol/l | 208.4 (74.8) | 230.8 (81.7) | <0.001 |
|  | Triglycerides in medium HDL | umol/l | 44.1 (10.1) | 47.8 (11.0) | <0.001 |
|  | Concentration of small HDL particles | nmol/l | 5013.0 (431.4) | 5158.2 (527.7) | <0.001 |
|  | Total lipids in small HDL | umol/l | 1114.4 (95.1) | 1145.2 (116.4) | <0.001 |
|  | Phospholipids in small HDL | umol/l | 601.6 (68.1) | 624.1 (84.7) | <0.001 |
|  | Free cholesterol in small HDL | umol/l | 115.9 (11.2) | 120.1 (13.9) | <0.001 |
|  | Triglycerides in small HDL | umol/l | 47.0 (10.8) | 51.4 (11.8) | <0.001 |
|  | Cholesterol esters in chylomicrons and extremely large VLDL | umol/l | 2.2 (1.6) | 2.6 (1.8) | 0.001 |
|  | Cholesterol esters in medium VLDL | umol/l | 84.2 (29.4) | 91.4 (30.7) | 0.001 |
|  | Triglycerides in very small VLDL | umol/l | 108.1 (28.9) | 115 (31.1) | 0.001 |
|  | Triglycerides in small LDL | umol/l | 32.9 (8.0) | 34.7 (8.6) | 0.003 |
|  | Total cholesterol in small VLDL | umol/l | 221.0 (55.2) | 232.3 (55.8) | 0.005 |
|  | Phospholipids in medium HDL | umol/l | 467.0 (61.5) | 480.0 (73.4) | 0.007 |
|  | Concentration of medium HDL particles | nmol/l | 2328.0 (318.7) | 2386.2 (381.3) | 0.02 |
|  | Triglycerides in IDL | umol/l | 122.5 (29.4) | 127.6 (31.5) | 0.02 |
|  | Triglycerides in large LDL | umol/l | 112.4 (26.0) | 116.8 (27.8) | 0.02 |
|  | Triglycerides in very large HDL | umol/l | 23.9 (7.1) | 25.1 (8.0) | 0.02 |
|  | Total lipids in medium HDL | mmol/l | 0.99 (0.14) | 1.01 (0.16) | 0.03 |
|  | Triglycerides in medium LDL | umol/l | 54.9 (12.8) | 57 (13.7) | 0.04 |
|  | Phospholipids in very large HDL | mmol/l | 0.37 (0.1) | 0.35 (0.1) | 0.04 |
|  | Cholesterol esters in small VLDL | umol/l | 135.9 (35.1) | 141.1 (35.2) | 0.05 |
| **Lipoprotein particle size** | Mean diameter for VLDL particles | nm | 36.3 (1.1) | 36.7 (1.2) | <0.001 |
|  | Mean diameter for HDL particles | nm | 10.3 (0.2) | 10.2 (0.2) | 0.02 |
| **Cholesterol** | Total cholesterol in VLDL | mmol/l | 0.69 (0.2) | 0.73 (0.2) | 0.001 |
|  | Total cholesterol in HDL3 | umol/l | 549.7 (28.2) | 554.1 (32.5) | 0.04 |
| **Glycerides and phospholipids** | Serum total triglycerides | mmol/l | 1.2 (0.4) | 1.4 (0.5) | <0.001 |
|  | Triglycerides in VLDL | mmol/l | 0.7 (0.3) | 0.9 (0.4) | <0.001 |
|  | Triglycerides in HDL | umol/l | 163 (31.8) | 174.3 (35.2) | <0.001 |
|  | Ratio of triglycerides to phosphoglycerides |  | 0.56 (0.15) | 0.61 (0.16) | <0.001 |
|  | Triglycerides in LDL | umol/l | 200.3 (46.4) | 208.5 (49.7) | 0.02 |
| **Amino acids** | Glutamine ‡ | mmol/l | 0.37 (0.1) | 0.35 (0.1) | <0.001 |
| Branched | Isoleucine | umol/l | 45.2 (12.4) | 48.9 (13.1) | <0.001 |
| Branched | Leucine | umol/l | 61.5 (13.5) | 65.8 (14.1) | <0.001 |
| Branched | Valine | umol/l | 129.9 (29.5) | 137.1 (28.6) | 0.001 |
| Aromatic | Phenylalanine | umol/l | 74.3 (9.9) | 76.8 (10.1) | 0.001 |
|  | Glycine | mmol/l | 0.22 (0.02) | 0.21 (0.02) | 0.03 |
| **Ketone body** | Acetoacetate | umol/l | 23.5 (10.3) | 27.0 (16.5) | <0.001 |
| **Fatty acids** | Monounsaturated fatty acids; 16:1, 18:1 | mmol/l | 3.8 (0.7) | 4.0 (0.8) | 0.001 |
|  | Ratio of omega-6 fatty acids to total fatty acids | % | 30.5 (2.3) | 29.9 (2.4) | 0.001 |
|  | Saturated fatty acids ‡ | mmol/l | 5.1 (0.7) | 5.3 (0.8) | 0.002 |
|  | Ratio of 18:2 linoleic acid to total fatty acids | % | 25.8 (2.7) | 25.2 (2.5) | 0.002 |
|  | Total fatty acids | mmol/l | 13.7 (2.0) | 14.1 (2.1) | 0.003 |
|  | Omega-3 fatty acids | mmol/l | 0.66 (0.13) | 0.70 (0.15) | 0.007 |
|  | Ratio of polyunsaturated fatty acids to total fatty acids | % | 35.3 (2.6) | 34.8 (2.8) | 0.01 |
|  | 22:6, docosahexaenoic acid | umol/l | 233.3 (44.4) | 241.1 (48.5) | 0.02 |
| **Inflammation** | Glycoprotein acetyls, mainly a1-acid glycoprotein | umol/l | 1465.8 (129.7) | 1520.4 (142.9) | <0.001 |
| **Glycolysis related metabolite** | Citrate ‡ | umol/l | 107.0 (16.3) | 111.0 (16.8) | 0.001 |
|  | Pyruvate | umol/l | 95.3 (40.1) | 102.9 (45.0) | 0.01 |
| **Fluid balance** | Albumin | umol/l | 83.6 (3.1) | 84.1 (4.0) | 0.03 |

GDM – gestational diabetes, VLDL – very large density lipoprotein, HDL – high density lipoprotein, IDL – intermediate density lipoprotein, LDL- low density lipoprotein

* p-value from Student’s t test or Mann Whitney test. Only metabolites associated with GDM (p<0.05) are reported.

† Mann Whitney test (median, IQR)

‡ missing data: glutamine n=1, saturated fatty acids n=1, citrate n=1
