# Supplementary material for: Early Antenatal Prediction of Gestational Diabetes in Obese Women: Development of Prediction Tools for Targeted Intervention
Source: PLoS One. 2016 Dec 8;11(12):e0167846. doi: 10.1371/journal.pone.0167846 (PMC5145208; doi:10.1371/journal.pone.0167846)
Supplement: S4 Table — (DOCX) [file pone.0167846.s004.docx]

S4 Table**. Calibration of Models 1–3**

| **Risk group** | **Number of participants** | **Number cases gestational diabetes** | **Predicted (mean)** | **Observed (mean)** |
| --- | --- | --- | --- | --- |
| **Model 1** |  |  |  |  |
| 0 - 10% | 110 | 5 | 0.08 | 0.05 |
| 11 - 20% | 425 | 69 | 0.15 | 0.16 |
| 21 - 30% | 334 | 84 | 0.24 | 0.25 |
| 31 - 40% | 193 | 70 | 0.34 | 0.36 |
| 41 - 50% | 100 | 40 | 0.44 | 0.40 |
| > 50% | 105 | 61 | 0.61 | 0.58 |
| **Model 2** |  |  |  |  |
| 0 - 10% | 127 | 7 | 0.06 | 0.06 |
| 11 - 20% | 189 | 31 | 0.15 | 0.16 |
| 21 - 30% | 162 | 40 | 0.25 | 0.25 |
| 31 - 40% | 105 | 31 | 0.35 | 0.30 |
| 41 - 50% | 86 | 43 | 0.45 | 0.50 |
| > 50% | 136 | 89 | 0.66 | 0.65 |
| **Model 3** |  |  |  |  |
| 0 - 10% | 132 | 6 | 0.06 | 0.05 |
| 11 - 20% | 167 | 29 | 0.15 | 0.17 |
| 21 - 30% | 152 | 41 | 0.25 | 0.27 |
| 31 - 40% | 112 | 31 | 0.35 | 0.28 |
| 41 - 50% | 68 | 33 | 0.45 | 0.49 |
| > 50% | 139 | 92 | 0.66 | 0.66 |
