# Supplementary material for: Early Antenatal Prediction of Gestational Diabetes in Obese Women: Development of Prediction Tools for Targeted Intervention
Source: PLoS One. 2016 Dec 8;11(12):e0167846. doi: 10.1371/journal.pone.0167846 (PMC5145208; doi:10.1371/journal.pone.0167846)
Supplement: S5 Table — (DOCX) [file pone.0167846.s005.docx]

S5 Table**. Exploring potential impact of missing data by examining clinical predictor associations with GDM on different sub-samples**

| **Variables** | **Maximal sample as used in**  **Model 1**  **(n=1267)**  **OR (95% CI)** | **Sub-sample that was used in**  **Model 2**  **(n=805)**  **OR (95% CI)** | **Sub-sample that was used in**  **Model 3**  **(n=770)**  **OR (95% CI)** |
| --- | --- | --- | --- |
| **Clinical** |  |  |  |
| Age (years) | 1.06 (1.03 - 1.09) | 1.06 (1.03 - 1.09) | 1.05 (1.01 - 1.08) |
| Previous GDM | 3.47 (1.45 - 8.30) |  |  |
| 1st degree relative T2DM | 1.40 (1.03 - 1.89) |  | 1.49 (1.02 - 2.16) |
| Sum of skinfold thicknesses (mm) | 1.01 (1.00 - 1.02) | 1.01 (1.01 - 1.02) | 1.01 (1.00 - 1.02) |
| Waist:height ratio (per 0.1) | 1.57 (1.25 - 1.98) | 1.37 (1.03 - 1.81) | 1.97 (1.29 - 3.01) |
| Neck:thigh ratio (per 0.1) | 1.55 (1.23 - 1.95) | 1.66 (1.25 - 2.21) | 2.50 (1.5 - 4.13) |
| Systolic BP (per 10 mmHg) | 1.36 (1.20 - 1.54) | 1.24 (1.08 - 1.44) | 1.26 (1.08 - 1.46) |
| Waist:thigh ratio (per 0.1) |  |  | 0.83 (0.69 - 0.99) |
| **AUC** | **0.71 (0.68 - 0.74)** | **0.68 (0.64 - 0.72)** | **0.69 (0.65 - 0.73)** |

GDM – gestational diabetes, OR – odds ratio, T2DM – type 2 diabetes mellitus, BP – blood pressure.
