# Supplementary material for: Early Antenatal Prediction of Gestational Diabetes in Obese Women: Development of Prediction Tools for Targeted Intervention
Source: PLoS One. 2016 Dec 8;11(12):e0167846. doi: 10.1371/journal.pone.0167846 (PMC5145208; doi:10.1371/journal.pone.0167846)
Supplement: S6 Table — (DOCX) [file pone.0167846.s006.docx]

S6 Table**. Sensitivity analysis for Models 1-3 excluding women with previous GDM**

|  | **Model 1**  **(n=1242)** | **Model 2**  **(n=791)** | **Model 3**  **(n=757)** |
| --- | --- | --- | --- |
|  | **OR (95% CI)** | **OR (95% CI)** | **OR (95% CI)** |
| **Clinical** |  |  |  |
| Age (years) | 1.05 (1.03 - 1.08) | 1.05 (1.01 - 1.08) |  |
| 1st degree relative T2DM | 1.39 (1.02 - 1.89) |  |  |
| Sum of skinfold thicknesses (mm) | 1.01 (1.01 - 1.02) | 1.01 (1.01 - 1.02) | 1.01 (1.01 - 1.02) |
| Waist:height ratio (per 0.1) | 3.45 (2.05 - 5.79) |  |  |
| Neck:thigh ratio (per 0.1) | 2.66 (1.73 - 4.08) | 1.55 (1.13 - 2.13) | 1.60 (1.15 - 2.23) |
| Waist:thigh ratio (per 0.1) | 0.75 (0.63 - 0.89) |  |  |
| Systolic BP (per 10 mmHg) | 1.22 (1.03 - 1.43) |  |  |
| Diastolic BP (per 10 mmHg) | 1.32 (1.05 - 1.65) | 1.36 (1.09 - 1.70) | 1.28 (1.01 - 1.61) |
| BMI (kg/m^2^) | 0.93 (0.88 - 0.98) |  |  |
| **Candidate biomarkers** |  |  |  |
| HbA1C (mmol/mol) |  | 1.11 (1.06 - 1.16) | 1.11 (1.05 - 1.16) |
| Random glucose (mmol/l) |  | 1.53 (1.22 - 1.91) | 1.77 (1.40 - 2.24) |
| Fructosamine (per 10umol/l) |  | 1.11 (1.02 - 1.20) | 1.12 (1.03 - 1.23) |
| Sex hormone binding globulin (per 10nmol/l) |  | 0.98 (0.96 - 0.99) | 0.97 (0.96 - 0.99) |
| Adiponectin (ug/ml)^a^ |  | 0.72 (0.59 - 0.88) | 0.73 (0.59 - 0.89) |
| Triglycerides (mmol/l)^a^ |  | 1.61 (1.07 - 2.41) |  |
| Ferritin (µg/l)^a^ |  | 1.17 (1.00 - 1.37) | 1.19 (1.01 - 1.40) |
| **Metabolome** |  |  |  |
| 3-Hydroxybutyrate (per 10umol/l) |  |  | 1.04 (1.01 - 1.07) |
| Trigs in medium HDL (per 10umol/l) |  |  | 1.61 (1.26 - 2.05) |
| Total cholesterol in HDL3 (per 10umol/l) |  |  | 1.14 (1.05 - 1.23) |
| Total fatty acids (mmol/l) |  |  | 0.86 (0.75 - 1.00) |
| **AUC** | **0.72 (0.69 - 0.75)** | **0.77 (0.73 - 0.80)** | **0.77 (0.74 - 0.81)** |

GDM – gestational diabetes, OR – odds ratio, T2DM – type 2 diabetes mellitus, BP – blood pressure, HbA1C - haemoglobin A1c, HDL – high density lipoprotein.

^a^Log transformed to base 2
